# 

**Published:** 1890-06

**Authors:** 


					﻿Entered at the Post Office, at Austin, Texas, as Second Class Mail Matter
Vol V
JUNE 1880
No. 12
DANIEL'S
MEDICAL
JOURNAL
Northern office, 1217 Filbert Street, Philadelphia.
Steam Book and Jor Printer, Austin, Texas
				

